# Using High Spatial Resolution to Improve BOLD fMRI Detection at 3T

**DOI:** 10.1371/journal.pone.0141358

**Published:** 2015-11-09

**Authors:** Juliana Iranpour, Gil Morrot, Béatrice Claise, Betty Jean, Jean-Marie Bonny

**Affiliations:** 1 UR370 QuaPA—INRA, F-63122, Saint-Genès-Champanelle, France; 2 Laboratoire Charles Coulomb—UMR 5221 CNRS, Université des Sciences et Techniques—Montpellier 2, place Eugène-Bataillon, 34090, Montpellier, France; 3 Neuroradiologie A, Plateforme Recherche IRM—CHU Gabriel-Montpied, F63000, Clermont-Ferrand, France; University of North Carolina, UNITED STATES

## Abstract

For different functional magnetic resonance imaging experiments using blood oxygenation level-dependent (BOLD) contrast, the acquisition of *T*_2_*-weighted scans at a high spatial resolution may be advantageous in terms of time-course signal-to-noise ratio and of BOLD sensitivity when the regions are prone to susceptibility artifacts. In this study, we explore this solution by examining how spatial resolution influences activations elicited when appetizing food pictures are viewed. Twenty subjects were imaged at 3 T with two different voxel volumes, 3.4 μl and 27 μl. Despite the diminution of brain coverage, we found that high-resolution acquisition led to a better detection of activations. Though known to suffer to different degrees from susceptibility artifacts, the activations detected by high spatial resolution were notably consistent with those reported in published activation likelihood estimation meta-analyses, corresponding to taste-responsive regions. Furthermore, these regions were found activated bilaterally, in contrast with previous findings. Both the reduction of partial volume effect, which improves BOLD contrast, and the mitigation of susceptibility artifact, which boosts the signal to noise ratio in certain regions, explained the better detection noted with high resolution. The present study provides further evidences that high spatial resolution is a valuable solution for human BOLD fMRI, especially for studying food-related stimuli.

## Introduction

With the widespread use of high magnetic fields, interest in increasing the spatial resolution in fMRI is constantly developing. While ultra-high field allows new levels of spatial resolution and specificity to be achieved [1, 2], it also makes sense to reduce the voxel volume (*V*) of gradient-echo echo-planar imaging (EPI) scans at less intense static fields, which are still those most commonly encountered. Some multi-resolution fMRI studies have indeed converged in demonstrating a better ability to detect neural activation in specific regions by acquiring high-resolution scans at 3 T; *e*.*g*. with *V* = 6.4–8.0 μl in amygdala [3, 4] or with *V* = 2.1 μl in brainstem [5].

The expected benefits of the voxel volume reduction are twofold. Firstly, it is a straightforward solution to mitigate BOLD-sensitivity modulations due to susceptibility artifacts [6–8]. When voxel size is reduced isotropically, its efficiency is furthermore not prone to the orientation of magnetic field gradients, known to change rapidly over the brain. Secondly, it may be advantageous in terms of time-course signal-to-noise ratio (tSNR) to acquire images with a reduced voxel volume, in which thermal noise dominates. In this regime, tSNR comes closer to what can be expected from the SNR of an individual image. Moreover tSNR increases steadily with the degree of smoothing, rather than being limited when physiological fluctuations with time dominate [9–11].

The voxel volume separating thermal noise and physiological noise dominance regimes has been determined experimentally: *V* = 5.8 μl for gray matter at 3 T with a 16-channel detector array head coil [12]. Thus it is suggested that a voxel volume far below the commonly used values, which are around 27 μl at 3 T, be used. Accordingly we compared activations elicited by viewing food pictures in two scanning conditions differing only in the voxel volume of acquisition at 3 T using two widely different isotropic volumes, 3.4 μl and 27 μl. Viewing food pictures is known to activate a large set of visual, gustatory and reward processing areas [13–27] that suffer to different degrees from susceptibility artifacts, such as occipital lobe, orbitofrontal cortex (OFC), amygdala or insula. Also, the results of two recent meta-analyses can provide useful normative data [28, 29] to assess the effect of voxel volume reduction, provided the examined experiments have been performed using a coarse voxel volume, corresponding unequivocally to a physiological noise dominant regime. In support, Table 1 records the voxel volume used in all the studies included in the meta-analyses, showing that *V* ≥ 27 μl for 20 out of 21 of them, and for all those at 3 T.

**Table 1:** Voxel volume, magnetic field strength and echo time of studies included in the meta-analyses [28, 29] on the neural correlates of processing visual food cues.

Reference	Voxel volume (μl)	Field (T)	Echo time (ms)
[30]	10.1	1.5	60
[17]	27.0	1.5	40
[31]	27.0	1.5	50
[32]	32.7	1.5	40
[33]	36.0	1.5	30
[34]	36.0	1.5	40
[22]	36.0	1.5	50
[14]	46.2	1.5	40
[23]	48.1	1.5	40
[27]	64.0	1.5	40
[15, 16]	67.3	1.5	40
**Mean**	**39.1**	**1.5**	**42.7**
[35]	27.0	3	30
[25]	27.0	3	40
[36]	27.0	3	30
[37]	33.1	3	30
[21]	36.0	3	30
[24]	54.9	3	25
[19, 20]	57.8	3	30
[18]	60.8	3	27
**Mean**	**40.4**	**3.0**	**30.3**

We hypothesized that the high spatial resolution (HR) protocol could reveal more significant and spatially-specific activations in response to viewing pictures of food in normal-weight subjects. In order to capitalize on the improved ability of high spatial resolution fMRI data to resolve fine spatial structures [38], it is necessary to adapt the width of the smoothing kernel to the true size of activation and to the contrast-to-noise ratio [39], both being *a priori* unknown. This is why both datasets were analysed using different smoothing sizes for selecting the appropriate coarser resolution in any anatomical region-of-interest (ROI).

Lastly, a 32-channel head coil was used in our study, which is theoretically more sensitive than the 16-channel one used by Bodurka *et al*. [12]. It is thus likely that the voxel volume that splits the thermal noise and physiological noise dominance regimes at 3 T may be lower than 5.8 μl for gray matter, *i*.*e*. rather close to the voxel volume used in the HR condition. That is why, to clarify the reasons for any improved activation detection, normalized maps of the baseline signal and of the noise were calculated at both voxel volumes. They were used to assess separately the effect of voxel volume reduction on the amplitudes of the susceptibility artifacts and of the physiological noise. Under these conditions, the question on how voxel size is affecting the sensitivity in BOLD-based fMRI at high field can be fully addressed.

## Methods

### Subjects

Twenty healthy, right-handed volunteers participated in this study (11 female, mean age ± SD = 25 ± 2 years; 9 male, mean age ± SD = 26 ± 2 years). All confirmed that they had no clinical history of major disease, and a normal eating behavior using the screening test for eating behavior disorders [40]. Hunger being known to modulate the response of certain areas to food pictures, the participants were food-restricted and were allowed to drink only water for 2 h before the fMRI session.

The study was approved by the ethics committee of the university hospital of Clermont-Ferrand (AU 993). Written informed consent from all the subjects was obtained before the experiment, in accordance with the Declaration of Helsinki.

### Stimulation paradigm

**Fig 1:**
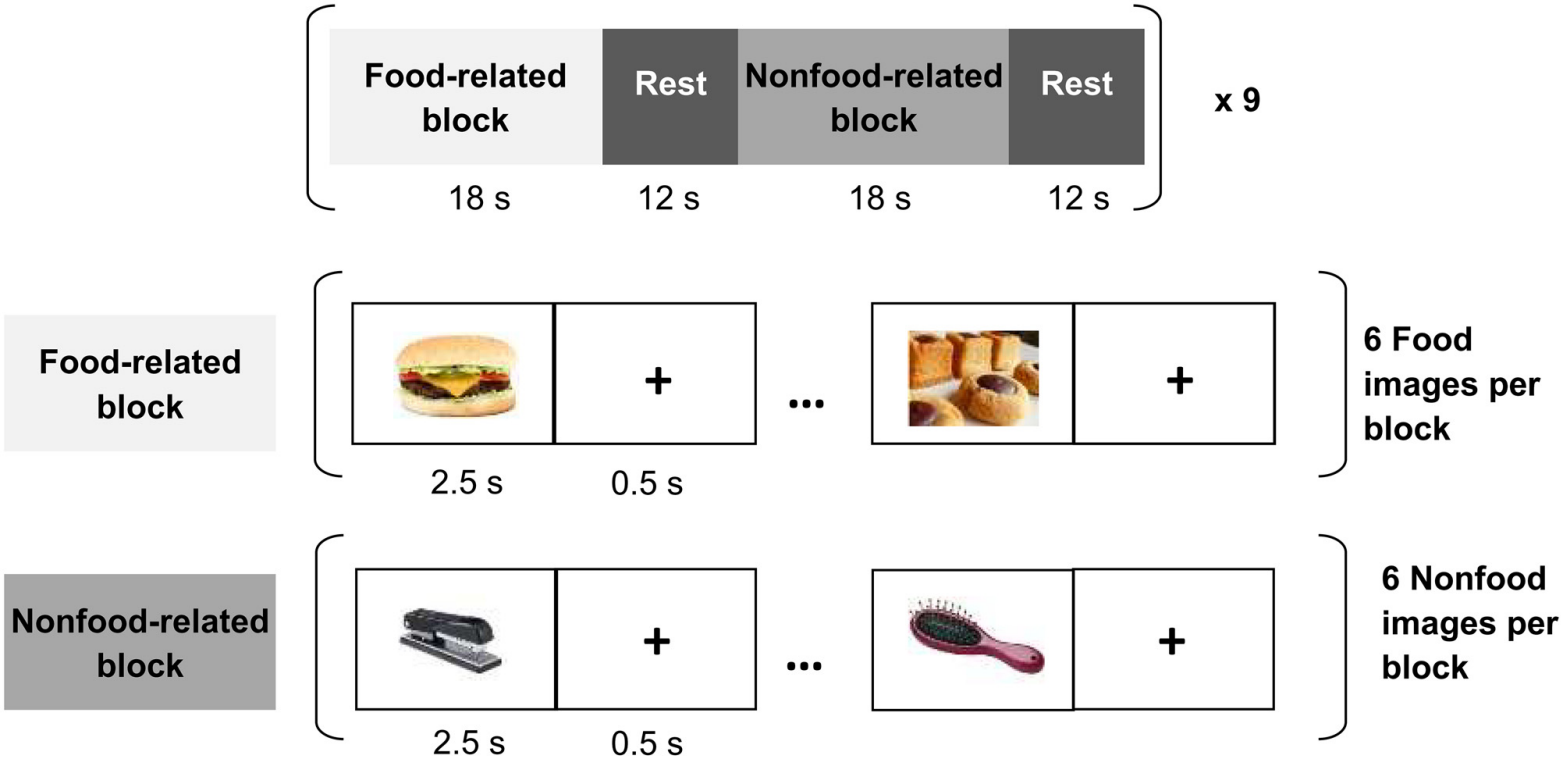
Graphical outline of the stimulation protocol used in this fMRI experiment. Stimuli were presented according to a block design involving food-related and non-food-related blocks. During the presentation of food images, participants were asked to imagine the taste of the viewed food, as if they were actually eating it. Each image was separated by a fixation cross and a rest period was placed between two blocks.

The stimulation paradigm consisted of alternating blocks presenting, in a randomized order, either ready-to-eat edible objects, or pictures of items that were clearly unrelated to food (see Fig 1). A fixation cross was presented in the center of the screen between two pictures in order to avoid saturation. In all, 54 different pictures were selected for each of the food-related and nonfood-related blocks. The food images depicted savory and sweet meals commonly served at breakfast, lunch and dinner. Images were presented to each of the subjects in randomized order. Pictures were presented using E-Prime 2.0 software (Psychological Software Tools, Sharpsburg, USA) via an MR-compatible visual stimulation system (NordicNeuroLab, Bergen, NO).

After an fMRI session, all the participants were asked to rate each picture showing food for motivational salience on a 7-point scale (0: extremely repulsive; 1: very repulsive; 2: quite repulsive; 3: neutral; 4: quite appetizing; 5: very appetizing; 6: extremely appetizing). The mean ± SD of these post-scanning ratings was 4.5 ± 1.2, significantly higher than 4 (paired Student’s *t*-test: *t* (54) = 12.2, *p* < 0.001), thus indicating that our subjects rated the pictures showing food as significantly appetizing.

### Data acquisition

The imaging data were collected on a 3 T General Electric Discovery MR750 MRI system (General Electric Medical Systems, Milwaukee, USA). A 32-channel receive-only phased-array head coil was used for brain imaging.

A *T*_1_-weighted inversion-recovery-prepared fast 3D gradient-echo sequence (BRAVO) was employed to obtain anatomical images of the whole brain of each subject. The acquisition parameters were: flip angle = 12°, inversion time = 400 ms, repetition time (TR) = 8.8 ms, echo time (TE) = 3.5 ms. The whole brain volume was covered at high-resolution using field-of-view = 240 × 192 × 175 mm^3^ and matrix 288 × 288 × 146 (*i*.*e*. voxel volume = 0.8 × 0.7 × 1.2 mm^3^, ∼ 0.7 μl).

Two functional experiments were conducted with the same paradigm, but differing in their voxel volume only. *T*_2_*-weighted gradient-echo images were collected using a 2D single-scan EPI sequence (TR = 3000 ms and flip angle = 90°). TE = 30 ms was chosen according to Table 1. Array spatial sensitivity encoding a parallel imaging option was activated for its ability to decrease the geometric distortion in EPI, the regions with inhomogeneous magnetic field being prone to such artifacts.

**Fig 2:**
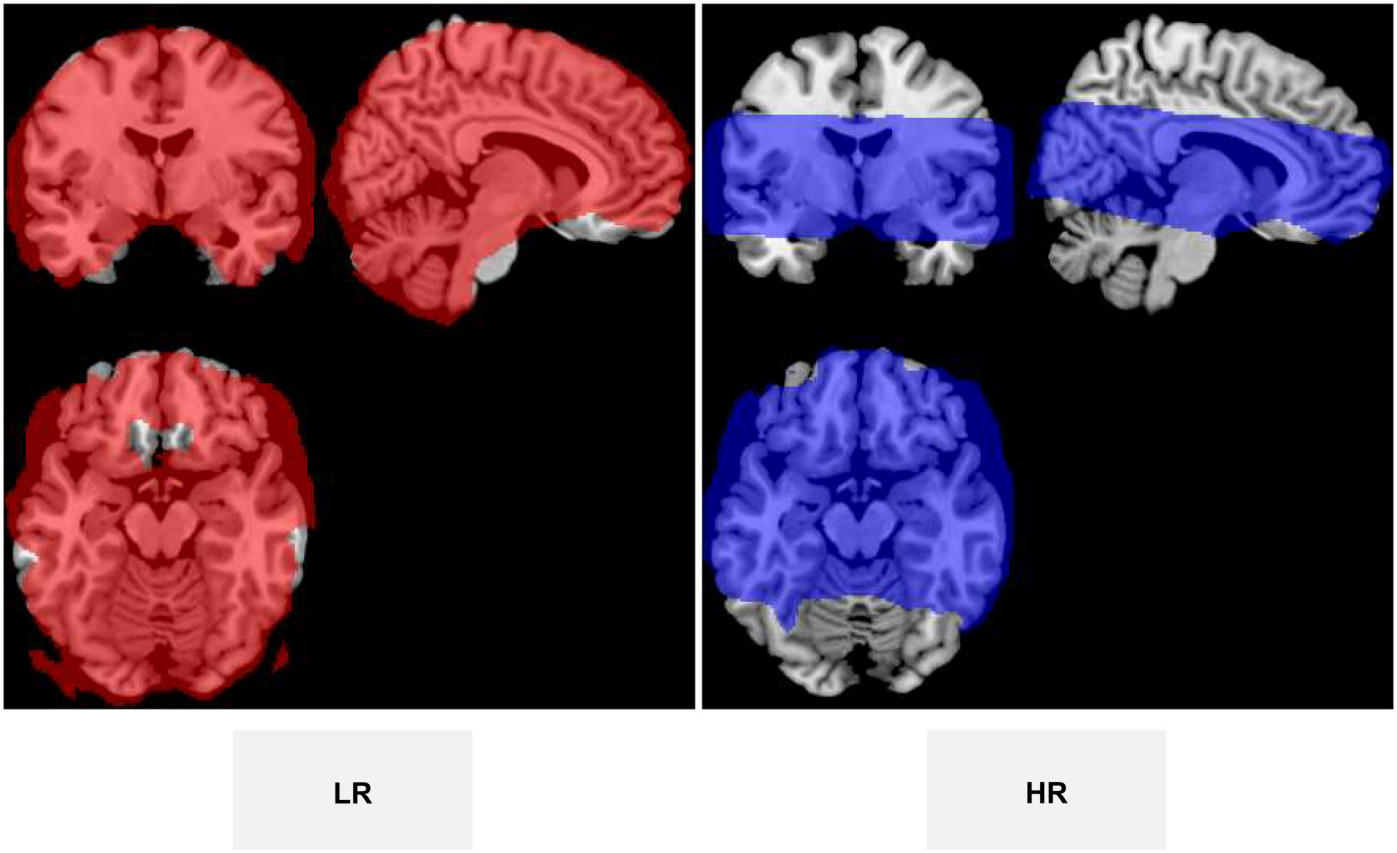
Masks showing the voxels contributing to the group analysis for the two conditions.

The low resolution (LR) dataset was obtained in axial orientation parallel to the anterior commissure-posterior commissure line with an isotropic 3 × 3 × 3 mm^3^ = 27 μl voxel volume (50 interleaved contiguous slices, field-of-view = 192 × 192 mm^2^, matrix = 64 × 64, reception bandwidth = 250 kHz, phase encoding along the anterior-posterior direction). The HR dataset was acquired in the same orientation with an 8 times lower voxel volume equal to 1.5 × 1.5 × 1.5 mm^3^ = 3.4 μl (42 interleaved contiguous slices, field-of-view = 192 × 192 mm^2^, matrix = 128 × 128, reception bandwidth = 250 kHz, phase encoding along the anterior-posterior direction). The 150 mm thick block was sufficient to cover the whole brain, but higher resolution with the same TR limited the coverage to 63 mm (see Fig 2). This acquisition volume ranged from *z* = −27 to 36 mm in Montreal Neurological Institute (MNI) space, which suffices for intercepting all brain regions known to be elicited when viewing food pictures [28, 29].

Each subject completed the same paradigm twice, the images being acquired in LR and HR conditions in a randomized order. Both consisted of the acquisition of 180 successive brain volumes.

### Data analysis

The fMRI datasets were preprocessed and analysed using SPM8 (Statistical Parametric Mapping, Wellcome Department of Cognitive Neurology, London, UK) implemented in Matlab (MathWorks Inc., Natick, USA). The analysis relative threshold *defaults*.*mask*.*thresh* (SPM default = 0.8) was decreased to 0.2 so that the voxels belonging to regions with signal dropout could contribute to the analysis whereas the background voxels were still excluded.

Images were firstly corrected for slice timing using the middle slice as reference. Secondly, images were realigned to the first image with a six-parameter rigid-body spatial transformation to correct for head motion. Thirdly, the anatomical scan was coregistered with the mean of realigned functional images after setting the origin of both the functional and the anatomical scans to the AC. The *New Segment* function was applied to segment anatomical images into gray matter, white matter and other tissues. The DARTEL warping method (high-dimensional Diffeomorphic Anatomical Registration Through Exponentiated Lie algebra) [41] was used to create flow fields specific to our subjects. The template was affine registered in MNI space. The functional images were normalized using compositions of flow fields (*i*.*e*. nonlinear deformations for warping all subject-specific images to the template) and template affine transformation parameters.

Fourthly, the normalized images of both LR and HR functional datasets were spatially smoothed with an isotropic Gaussian kernel of different sizes, expressed by the full width half-maximum (FWHM) in mm. FWHM ranged from 1.5 mm to 8 mm for the HR dataset, from 2.5 mm to 8 mm for the LR one, with a step of 0.5 mm. The maximum FWHM corresponds to 2−3 times the voxel size of the LR dataset as usually chosen [42].

For each subject, conditions and Gaussian kernel sizes (*i*.*e*. 20 x 2 x (12 + 14) = 1040 datasets), first-level statistical parametric maps were first generated using the general linear model to describe the variability of the data on a voxel-by-voxel basis. The model consisted of a boxcar function, using the food-related and non-food-related blocks as regressors of interest, convolved with the canonical SPM hemodynamic response function. The contrast between viewing food and non-food pictures was then generated.

Subsequently, a second-level group random effects analysis was performed to subject this contrast to a one-sample *t*-test. A cluster was chosen for its significance in two steps; using a primary voxel-level thresholding at a level *p*_*p*_ and by evaluating the cluster-level FDR-corrected *p*-value (*Q*_FDR_), which gives the family-wise error rate probability (*i*.*e*. the level of false-positives in the cluster) due to multiple comparisons [43]. *p*_*p*_ was set to 0.001 to avoid false positives and lack of specificity [44] and a cluster was considered as significant when *Q*_FDR_ was less than 0.05. No minimum size of contiguous voxels was required.

The obtained clusters were inspected in several anatomical ROIs specified by the Automated Anatomical Labeling (AAL) atlas [45]. The most concurrent regions activated in response to viewing food pictures were selected according to the meta-analyses [28, 29], *i*.*e*. fusiform gyrus, middle and superior occipital gyrus, lingual gyrus, lateral OFC (Frontal Mid Orb), insular cortex and amygdala. The parietal gyrus was not investigated because this region was outside the covered block in HR condition.

For analysing the effect, smoothing and selecting an appropriate FWHM in a given ROI, the selected value of FWHM was the lowest leading to a significant cluster. This choice is justified by the concern of obtaining less extensive clusters, so better localized, and prevents regions that are functionally different from merging together [46].

For further analysis at the individual level of the sensitivity differences between the two conditions LR and HR, three metrics were evaluated in several anatomical ROI. Firstly, the 95^th^ percentile measures of the percent signal change (PSC) was calculated in order to quantify a BOLD variation representative of the typically active voxels within the ROI. Secondly, the baseline mean signal and the standard deviation of noise were also mapped from the 42 images of the EPI time-series acquired during the rest periods. To neutralize the between-subject changes of mean signal intensity, these individual maps were then intensity-normalized using the signal of the cerebral peduncle (delineated by the JHU white-matter atlas) as a reference. Finally, baseline mean signal and standard deviation of noise were averaged within each anatomical ROI.

To compare the means of PSC, baseline signal and noise obtained in the two conditions, paired-sample two-tailed Student’s *t*-test or Wilcoxon signed-rank tests were applied according to a prior Shapiro test for normality. All these statistical analyses were carried out with the open source R Studio Software (http://www.rstudio.com/).

## Results

**Fig 3:**
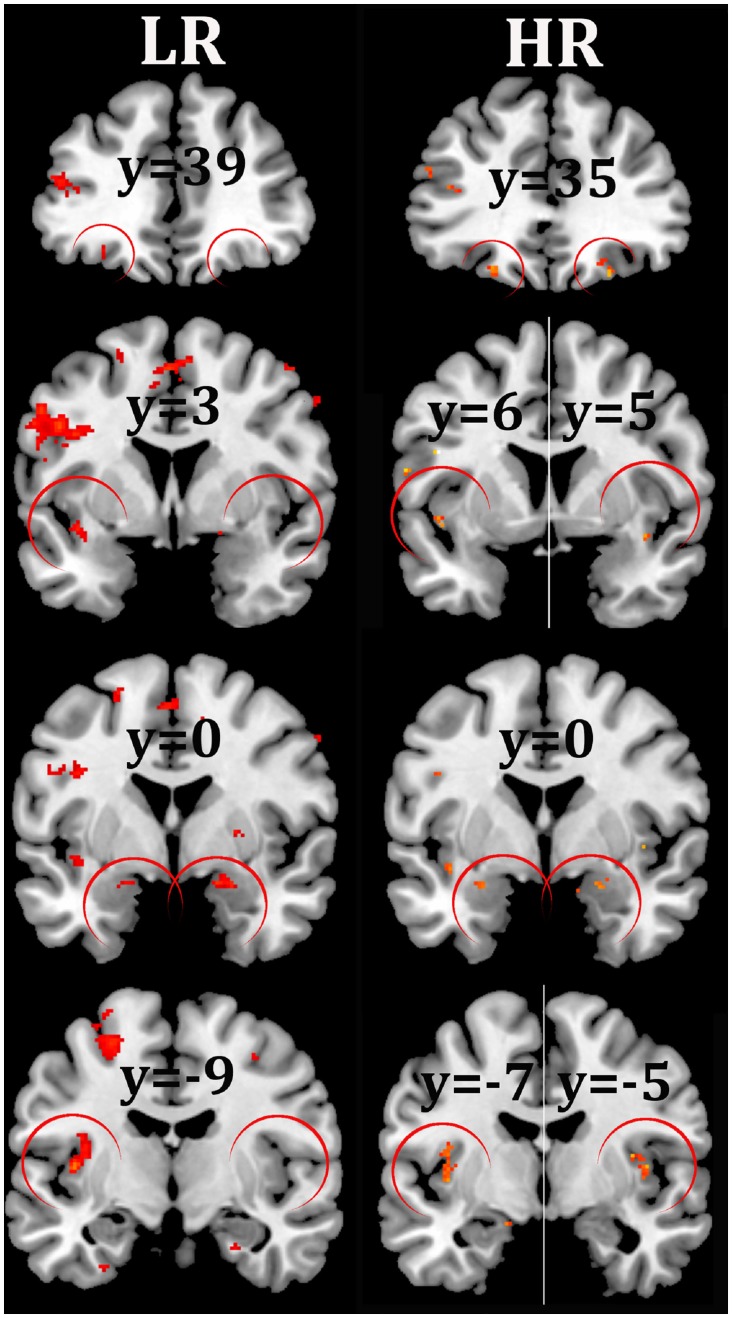
Group statistical parametric maps for the comparison of significantly activated regions detected under LR and HR conditions for the contrast between viewing food and non-food pictures. Activations were successively shown in the OFC (y = 35/39), anterior insula (y = 3/6), amygdala (y = 0) and insula (y = -5/-9) using a voxel-wise p<0.001 uncorrected threshold, with an extent threshold of 5 voxels (neurologic orientation, right-on-right). Under such conditions, the activations observable in the left amygdala and right OFC with LR do not resist to the *Q*_FDR_ < 0.05 threshold used in Table 2.

The brain regions activated at the group level for the contrast between viewing food and nonfood pictures are represented in Table 2 for both LR and HR conditions, and visually compared in Fig 3. For the same significance threshold (*Q*_FDR_ < 0.05), more regions were found significantly activated with the HR imaging protocol than with the LR one. Bilateral activation of the lateral OFC was detected only in the HR condition. Moreover the insula and amygdala that were significantly activated unilaterally in the LR condition, reached bilateral significance in the HR condition (Table 2, Fig 3).

**Table 2:** Locations (MNI) of activated brain regions at the group level for the contrast between viewing food and non-food pictures obtained from HR data. The reported clusters were thresholded at the same *p* < 0.001 (uncorrected for multiple comparisons). *Q*_FDR_ indicates the level of FDR on clusters.

Brain region	HR						LR					
	Brodmann area	Volume (K_E_)	x, y, z (mm)	t	Q_FDR_	Smoothing (mm)	Brodmann area	Volume (K_E_)	x, y, z (mm)	t	Q_FDR_	Smoothing (mm)
**L Amygdala**	34	9	-26, 0, -18	4.26	0.039	1.5	-	-	-	-	-	-
	34	14	-20, -3, -18	4.69	0.008	1.5	-	-	-	-	-	-
**R Amygdala**	34	20	23, 2, -17	5.69	0.005	1.5	34	51	18, 0, -17	5.12	0.016	2.5
**L Frontal Mid Orb**	11	29	-23, 35, -18	4.97	0.000	1		-	-	-	-	-
**R Frontal Mid Orb**	11	6	26, 35, -21	5.43	0.045	1		-	-	-	-	-
**L Insula**	13	61	-36, -10, 13	5.45	0.000	1	13	140	-41, -9, 6	6.38	0.000	2.5
	38	7	-44, 6, -8	4.95	0.029	1	13/38	42	-39, 3, -11	4.79	0.03	2.5
	13/22	11	-44, 3, -2	4.83	0.006	1		-	-	-	-	-
	13	6	-39, 0, -11	4.22	0.045	1		-	-	-	-	-
**R Insula**	13	27	40, -3, 4	5.74	0.000	1		-	-	-	-	-
	13	7	39, 5, -12	5.1	0.029	1		-	-	-	-	-
	38	9	45, 12, -8	4.86	0.013	1		-	-	-	-	-
**L Fusiform**	19	20	-29, -75, -9	4.65	0.000	1	19	330	-27, -61, -15	6.91	0.000	2.5
		-	-	-	-	-	19	35	-29, -57, -8	5.51	0.003	2.5
		-	-	-	-	-	19	16	-24, -51, -14	4.75	0.035	2.5
**R Fusiform**	19	15	27, -52, -11	5.56	0.006	1	19	968	23, -78, -14	8.54	0.000	2.5
**L Lingual**	17/18	12	-14, -88, -5	4.69	0.004	1	17/18	1315	-5, -82, -2	9.1	0.000	2.5
**R Lingual**	17	19	11, -88, -3	5.71	0.005	1.5	17/18	1312	11, -84, -11	11.36	0.000	2.5
		-	-	-	-	-	19	68	29, -54, -6	6.1	0.007	2.5
**L Occ mid**	18/19	71	-29, -82, 12	7.31	0.000	1		-	-	-	-	-
	18/19	108	-18, -96, 7	6.19	0.000	1	18/19	1240	-17, -96, 6	7.99	0.000	2.5
	18/19	12	-38, -78, 4	5.78	0.004	1		-	-	-	-	-
	18/19	56	-29, -82, 1	5.68	0.000	1		-	-	-	-	-
	18/19	9	-24, -73, 25	5.5	0.013	1		-	-	-	-	-
**R Occ mid**	18/19	59	34, -85, 12	5.58	0.000	1		-	-	-	-	-
	18/19	7	28, -91, 7	4.79	0.029	1	18/19	694	27, -94, 10	8.22	0.000	2.5
	18/19	6	24, -93, 4	4.77	0.045	1		-	-	-	-	-
**L Occ sup**	17	55	-11, -96, 1	5.72	0.000	1	17/18	309	-15, -93, 4	7.15	0.000	2.5
	19/7	36	-20, -69, 37	5.23	0.000	1	19/7	132	-26, -67, 39	5.86	0.000	2.5
		-	-	-	-	-	19/7	143	-23, -76, 42	7.58	0.000	2.5
**R Occ sup**	18	13	23, -88, 10	4.77	0.004	1	18/19	244	26, -94, 12	7.79	0.000	2.5
		-	-	-	-	-	19/7	211	27, -70, 37	5.86	0.000	2.5

The fMRI activation metrics are listed in Table 3 for the two conditions LR and HR. Essentially, this showed that PSC is greater under HR conditions for all the studied regions except the right lateral OFC (Frontal Mid Orb). The baseline signal is significantly stronger under HR in the mygdala and the OFC, while the noise is weaker under HR only in the left OFC.

**Table 3:** Metrics characterizing the fMRI activations at the individual level obtained in the two conditions of acquisition, LR and HR.

Brain region	t-test (PSC)	PSC				t-test (baseline)	Baseline				t-test (noise)	Noise			
		HR		LR			HR		LR			HR		LR	
		Mean	SD	Mean	SD		Mean	SD	Mean	SD		Mean	SD	Mean	SD
**L Amygdala**	p < 0,001	1.57	0.49	1.13	0.60	p < 0,05	1.23	0.08	1.14	0.12	NS	1.10	0.19	1.10	0.16
**R Amygdala**	p < 0,01	1.34	0.41	1.01	0.45	p < 0,05	1.17	0.12	1.11	0.12	NS	1.18	0.20	1.21	0.26
**L Frontal Mid Orb**	p < 0,005	2.20	0.90	1.57	0.76	p < 0,0001	1.74	0.41	1.44	0.42	p < 0,001	1.83	0.59	2.29	0.80
**R Frontal Mid Orb**	NS	2.36	1.09	2.15	1.21	p < 0,0001	1.45	0.35	1.19	0.36	NS	1.72	0.60	1.85	0.66
**L Insula**	p < 0,0001	1.21	0.20	0.68	0.27	NS	1.60	0.13	1.61	0.13	NS	1.37	0.36	1.33	0.25
**R Insula**	p < 0,0001	1.27	0.26	0.67	0.30	NS	1.63	0.14	1.62	0.13	NS	1.46	0.31	1.47	0.23

## Discussion

In this fMRI study, we examined the characteristics of brain activations elicited by viewing food cues compared with non-food ones using two imaging protocols that differ only in the voxel volume of the scans. These kinds of paradigm are known to lead to a low inter-study reproducibility since only 12 to 41% of the experiments contributed to the clusters [29].

In conditions of similar resolutions (LR), the activations detected in our study recover only a part of the activations described by the meta-analyses [28, 29], in particular no activation was found in left amygdala, left lateral OFC, and right insula. A lack of sensitivity undoubtedly explains the low inter-study reproducibility reported in the meta-analyses and justifies using the possibly more sensitive conditions of HR.

All the activations reported in the meta-analyses occurred under our HR conditions, at a high level of statistical significance. The *Q*_FDR_ found was often much lower than the conservative threshold of 0.05. This point deserves to be emphasised since the activations revealed by meta-analysis are generally more robust, being less prone to false alarms, because these errors will not be replicated across studies [47].

In addition, these results show that activations that are unilateral in the meta-analyses are found as bilateral under HR conditions. Firstly, the lateral OFC (Frontal Mid Orb) which was found activated on the left [MNI (−26, 32, −14) in [29] and MNI (−25, 31, −17) in [28]] was bilaterally activated in our HR condition [MNI (−23, 35, −18) and (26, 35, −21)]. The lateral OFC is presumed to provide a value representation, regardless of stimulus modality, and even for stimuli that were merely imagined [48–51]. That is why the lateral OFC responds to viewing rewarding food pictures, but the detection of any activity is difficult because of the close air-tissue interfaces. The magnetic susceptibility differences between air and soft tissues create magnetic field gradients around the frontal sinuses, and thus signal dropouts [52, 53]. Other drawbacks come from the poor quality of spatial normalization due to more marked spatial distortions, or the spatial variability of stimulus-specific responses, reflecting inter-subject differences of the affective value when viewing the food images [54]. It is worth noting that the right activation is much weaker than the left one (*i*.*e*. reduced volume and higher *Q*_FDR_). It is therefore likely that the bilateral detection may occur because of the increase in BOLD sensitivity due to the HR condition. A quite similar point is the finding that bilateral activation is restored using HR in the amygdala, while the meta-analyses report only the activation in the left side. The amygdala is known to play a role in reward processing, and its activation by appetizing food images has been observed in subjects with enhanced motivation due to hunger [14, 18]. As for the lateral OFC, it is known that the detection of activation in this region is complicated by the presence of a magnetic field gradient [55]. Our experimental results confirm the earlier suggestion of Merboldt *et al*. [56] that reliable BOLD fMRI of the amygdala requires voxel sizes of 4–8 μl or less. Finally, our study emphasizes that food images in fact elicit bilateral activations in the brain, and that acquiring HR data allows such patterns to be revealed.

It has been shown in [29] that the activations mainly occurred in the left hemisphere. Our results are in line with this observation, and show that the number of significantly activated voxels reaches ~66% in the left hemisphere under both LR and HR conditions. At first sight, this seems to support the valence asymmetry hypothesis of emotion, which posits that the left hemisphere is dominant for positive and the right for negative emotions [57]. Nevertheless, there has not so far been clear-cut evidence for a systematic left-dominance, the region considered [58] and factors such as gender [59] influencing the dominance. Furthermore, the lateralization of activations may also be influenced by methodological artifacts, as previously observed in amygdala [60].

Activations of both the middle and the anterior parts of the insular cortex in response to viewing food pictures were detected under both conditions, but only HR allowed activation of the insular cortex to be observed bilaterally. The first cluster obtained with HR intercepts the part of the anterior insula which overlies the frontal operculum. The second one shows two distinct parts in the middle insula which overlies the Rolandic operculum. Notably, this corresponds with the precise description of gustatory representation within insula obtained by meta-analysis [48]. The activation of the insula found is in line with its responsiveness to most of the food-related stimuli. In addition to the representation of the gustatory aspects of intra-oral stimuli, the insular taste cortex may have other small functions [61, 62], such as evaluating the biological significance of these stimuli [63]. In sum, finding a bilateral activation in HR of both the posterior and the anterior parts is not surprising, but attributing specific functions to the activated sub-regions remains doubtful.

The activation of the visual system is explained by a stronger elicitation by food than by non-food images, probably because of a greater attentional or motivational salience of food objects (18, 19).

At the individual level, our results (Table 3) emphasize, firstly, that the PSC is significantly higher under HR condition, probably due to the partial volume effect. Indeed, the mixing of active tissues with non-active ones is less likely for a lower voxel volume. In addition, the significant increases of the baseline signal are explained by the reduction of inhomogeneity-induced signal de-phasing due to smaller voxels. This interpretation is corroborated by previous studies which reported the singular amplitude of susceptibility gradients in amygdala [64] and in OFC [65]. We do not highlight important differences of noise levels between the conditions (only a unilateral reduction in the lateral OFC). A 32-channel head coil was used in our study, which is theoretically more sensitive than the 16-channel one used by Bodurka *et al*. [12]. It is thus likely that the voxel volume that splits the thermal noise and physiological noise dominant regimes at 3 T in our experimental conditions may be lower than 5.8 μl for gray matter. Hence the physiological noise may still be dominant using a voxel volume of 3.4 μl, which could explain the slightly different noise levels when comparing LR and HR conditions.

We observe also at the individual level that the across-subject variance on the volume of activation were salient, in agreement with the previous results obtained from a test-retest experiment [66]. This poor reproducibility doubtlessly explains why the mean volume of the activations did not significantly differ between the two conditions LR and HR (data not shown). However, comparing these volumes between individuals, a significant correlation between the two conditions was found, which confirms a reduction in the volume of activations in HR relative to LR, probably because of a lower partial volume effect. Our study indicates that the improvement of sensitivity due to smaller voxel volume has multiple causes and is region-dependent. Indeed, the SNR enhancement due to the mitigation of susceptibility artefact is significant only in regions where the amplitude of magnetic field gradients leads to substantial signal loss (*e*.*g*. OFC). In regions not prone to such losses, the sensitivity of PSC to the partial volume effect is sufficient justification for acquiring data in HR, especially when the CNR is low. Moreover, the HR condition offers a greater possibility of adapting the width of the smoothing kernel to the true size of activation and to the contrast-to-noise ratio (31), both being unknown. Using HR data and small smoothing kernel width, our results highlight rather small but bilateral and significant clusters in the lateral OFC, the insula and the amygdala (see Table 2 and Fig 3). Most of these activations were observed at HR overlap with already identified taste-responsive regions [48], which supports the involvement of the gustatory cortex when viewing food images, *i*.*e*. even in the absence of a chemosensory stimulus.

Because many voxels are not prone to vascular effects at 3T and thus have spatial specificity matching the voxel size [2], we can be quite confident about the location of the obtained clusters. Our results suggest that it would be appropriate to reduce further the volume of voxels for obtaining images less prone to physiological noise throughout the brain. However, with single-shot 2D EPI, increasing the spatial resolution lengthens the time necessary to cover a given brain volume. This can be explained (i) by the lengthening of the sampling trajectory, which must traverse an enlarged k-space, and (ii) by the reduction of the slice thickness, which requires a corresponding increase in the number of slices needed. The simplest solution is to increase the acquisition time, which has the drawback of reducing the density of the temporal sampling. Many other solutions have been developed to improve the spatial resolution without having to degrade the temporal resolution, such as simultaneous multi-slice excitation with multiband radiofrequency pulses [67], parallel imaging [68] or partial-Fourier acquisition [69]. We can expect these developments to continue in this domain owing to constantly improving sensitivity, which allows simultaneous advance in sensitivity and in functional specificity.

## Conclusion

Our results demonstrate that acquisition with a voxel volume of 3.4 μl at 3 T leads to a better detection of activations in response to viewing pictures of food compared with the common voxel volume of 27 μl. On the basis of a single group-study using 20 subjects, the regions in which the activations were detected using high spatial resolution gradient-echo EPI were notably consistent with those reported in two activation likelihood estimation meta-analyses. Furthermore, frontal and temporal taste-responsive regions (*i*.*e*. OFC, amygdala), known to suffer from severe susceptibility artifacts, were found activated bilaterally, that contrasts with previous findings. Such sensitive detection was obtained by optimizing the smoothing size to take more account of partial volume effects, which greatly affect fMRI performance.
